# Supplementary material for: Unified control of amoeboid pseudopod extension in multiple organisms by branched F-actin in the front and parallel F-actin/myosin in the cortex
Source: PLoS One. 2020 Dec 9;15(12):e0243442. doi: 10.1371/journal.pone.0243442 (PMC7725310; doi:10.1371/journal.pone.0243442)
Supplement: S1 Table — (PDF) [file pone.0243442.s001.pdf]

S1 table. Source of movies.

strain	reference	remarks
WT, polarized	(1–3)	main source of movies
	(4–6)	WT control of mutant studies
WT, unpolarized	(2,4,7)	
WT, chemotaxis	(3)	
WT, under agar	(8)	
scar-null	(4)	movies used here
		see also Ura et al (9)
pla2-null	(1)	
gc-null	(1)	
gbpC-null	(5,10)	
myoII-null	(4)	
Rap1G12V	(11)	similar to movie S1,2 at 1 second per frame
forAEH-null	(12)	
racE-null	(12)	
lrrA-null	(13)	
Neutrophils	(14)	
	(15)	movie from the Babraham Institute; downloaded on May 6, 2015.
Mesenchymal cells	(16)	movie from the Institut fur Technische Chemie, Hannover; downloaded on May 6, 2015.
		reference to authors of the movie (17)
B.d. chytrid	(18)	
WT, electrotaxis	(19)	

WT: wild-type *Dictyostelium*

References

1. Bosgraaf L, Van Haastert PJM. The ordered extension of pseudopodia by amoeboid cells in the absence of external cues. PLoS One. 2009 Jan;4(4):e5253.

2. Van Haastert PJM, Bosgraaf L. Food searching strategy of amoeboid cells by starvation induced run length extension. PLoS One. 2009 Jan;4(8):e6814.

3. Bosgraaf L, Van Haastert PJM. Navigation of chemotactic cells by parallel signaling to pseudopod persistence and orientation. PLoS One. 2009 Jan;4(8):e6842.

4. van Haastert PJM, Keizer-Gunnink I, Kortholt A. The cytoskeleton regulates symmetry transitions in moving amoeboid cells. J Cell Sci. 2018 Apr;131(7).

5. Kortholt A, van Egmond WN, Plak K, Bosgraaf L, Keizer-Gunnink I, van Haastert PJM. Multiple regulatory mechanisms for the Dictyostelium Roco protein GbpC. J Biol Chem. 2012 Jan;287(4):2749–58.

6. Van Haastert PJM, Bosgraaf L. The local cell curvature guides pseudopodia towards chemoattractants. HFSP J. 2009 Aug;3(4):282–6.

7. Van Haastert PJM. A model for a correlated random walk based on the ordered extension of pseudopodia. PLoS Comput Biol. 2010 Aug;6(8).

8. Van Haastert PJM. Amoeboid cells use protrusions for walking, gliding and swimming. PLoS One. 2011;6(11):e27532.

9. Ura S, Pollitt AY, Veltman DM, Morrice NA, Machesky LM, Insall RH. Pseudopod growth and evolution during cell movement is controlled through SCAR/WAVE dephosphorylation. Curr Biol. 2012 Apr;22(7):553–61.

10. Veltman DM, van Haastert PJM. The role of cGMP and the rear of the cell in Dictyostelium chemotaxis and cell streaming. J Cell Sci. 2008 Jan;121(Pt 1):120–7.

11. Plak K, Keizer-Gunnink I, van Haastert PJM, Kortholt A. Rap1-dependent pathways coordinate cytokinesis in Dictyostelium. Mol Biol Cell. 2014 Dec 15;25(25):4195–204.

12. Litschko C, Bruhmann S, Csiszar A, Stephan T, Dimchev V, Damiano-Guercio J, et al. Functional integrity of the contractile actin cortex is safeguarded by multiple Diaphanous-related formins. Proc Natl Acad Sci U S A. 2019 Feb;116(9):3594–603.

13. Kamp M. Regulation of G-protein during chemotaxis in space and time. PhD thesis, Groningen Univ. 2019;

14. Itakura A, Aslan JE, Kusanto BT, Phillips KG, Porter JE, Newton PK, et al. p21-Activated kinase (PAK) regulates cytoskeletal reorganization and directional migration in human neutrophils. PLoS One. 2013;8(9):e73063.

15. https://www.youtube.com/watch?v=qOX5Z7AutM4.

16. https://www.youtube.com/watch?v=RRUvRJNlW84.

17. Kirsch M, Birnstein L, Pepelanova I, Handke W, Rach J, Seltsam A, et al. Gelatin-Methacryloyl (GelMA) Formulated with Human Platelet Lysate Supports Mesenchymal Stem Cell Proliferation and Differentiation and Enhances the Hydrogel's Mechanical Properties. Bioeng (Basel, Switzerland). 2019 Aug;6(3).

18. Fritz-Laylin LK, Lord SJ, Mullins RD. WASP and SCAR are evolutionarily conserved in actin-filled pseudopod-based motility. J Cell Biol. 2017 Jun;216(6):1673–88.

19. Zhao M, Jin T, McCaig CD, Forrester J V, Devreotes PN. Genetic analysis of the role of G protein-coupled receptor signaling in electrotaxis. J Cell Biol. 2002 Jun;157(6):921–7.
