# Supplementary material for: Unified control of amoeboid pseudopod extension in multiple organisms by branched F-actin in the front and parallel F-actin/myosin in the cortex
Source: PLoS One. 2020 Dec 9;15(12):e0243442. doi: 10.1371/journal.pone.0243442 (PMC7725310; doi:10.1371/journal.pone.0243442)
Supplement: S2 Table — (PDF) [file pone.0243442.s002.pdf]

Table S2. Overview of all data

strain		basic properties				START kinetics		STOP kinetics				distribution		pseudopods per cell			calculated parameters								
		size (S) µm	time (T) sec	rate (v) µm/s	interval sec	α1 fraction/s	A	γ fraction/s2	kv s/µm	ks 1/µm	kt 1/s	$\alpha_1/\tilde{\beta}$	A	extending	total	ext/total (%)	A mean	$\tilde{\beta}$ (fraction/s) $\alpha/(\alpha/\tilde{\beta})$	$\sqrt{0.5\gamma}$	$0.5\sqrt{\pi}/T$	**	mean	contributions to STOP (%) rate (kv*v)	size (ks*s)	time (kt*t)
Dictyostelium WT, polarized	mean	5.369	11.602	0.549	15.418	0.140	3.586	0.01240	0.085	0.132	0.023	1.791	3.548	0.845	2.109	40%	3.567	0.0780	0.0787	0.0764	0.0885	0.0804	4.6%	69.2%	26.2%
996 pseudopods,	95% CI	0.126	0.372	0.018	0.706	0.005	0.280	0.00035	0.039	0.018	0.005	0.133	0.316	0.010	0.174	3%	0.298	0.0064	0.0044	0.0024	0.0241	0.0047	1.0%	4.8%	2.7%
39 cells, 10 movies	n	996	996	996	957	14	14	21	996	996	996	996	996	15356	101	101	14	14	21	996	996	21	996	996	996
WT, unpolarized	mean	4.896	11.084	0.526	16.885	0.142	3.662	0.01490	0.011	0.153	0.016	1.700	5.110	0.774			4.386	0.0834	0.0863	0.0800	0.0857	0.0839	0.6%	80.6%	18.7%
344 pseudopods,	95% CI	0.176	0.612	0.028	1.385	0.005	0.352	0.00052	0.095	0.032	0.007	0.070	0.025	0.016			0.188	0.0047	0.0061	0.0044	0.0533	0.0086	2.7%	8.8%	4.1%
14 cells, 6 movies	n	344	344	344	330	14	14	21	344	344	344	344	344	5808			14	14	21	344	344	21	344	344	344
WT, chemotaxis	mean	5.374	11.377	0.553	14.235	0.167	4.164	0.01344	0.100	0.139	0.018	1.837	3.961	0.796			4.062	0.0910	0.0820	0.0779	0.0889	0.0850	5.5%	74.1%	20.4%
414 pseudopods,	95% CI	0.204	0.580	0.027	1.043	0.005	0.640	0.00063	0.076	0.019	0.005	0.292	1.118	0.016			0.879	0.0147	0.0077	0.0040	0.0442	0.0088	2.1%	5.5%	3.1%
14 cells, 6 movies	n	414	414	414	400	14	14	21	414	414	414	414	414	5893			14	14	21	414	414	21	414	414	414
WT, under agar	mean	4.624	7.592	0.715	7.820	0.355	3.090	0.03209	0.427	0.120	0.022	2.556	4.281	0.953			3.685	0.1389	0.1267	0.1167	0.1374	0.1299	29.7%	54.0%	16.3%
218 pseudopods,	95% CI	0.270	0.592	0.043	0.729	0.035	0.713	0.00183	0.130	0.020	0.010	0.068	0.120	0.031			0.417	0.0144	0.0144	0.0091	0.0967	0.0168	4.6%	4.9%	3.7%
7 cells, 4 movies	n	218	218	218	211	10	10	12	218	218	218	218	218	1704			10	10	12	218	218	12	218	218	218
scar-null	mean	1.430	2.356	0.790	3.556	0.363	4.293	0.30720	0.100	0.595	0.062	0.966	3.676	0.617			3.985	0.3755	0.3919	0.3762	0.5122	0.4140	7.3%	79.1%	13.6%
251 pseudopods,	95% CI	0.062	0.179	0.045	1.170	0.047	0.510	0.10510	0.039	0.151	0.016	0.001	0.010	0.033			0.260	0.0488	0.2682	0.0286	0.1274	0.0591	1.4%	10.5%	1.8%
6 cells, 3 movies	n	251	251	251	245	9	9	6	251	251	251	251	251	892			9	9	6	251	251	6	251	251	251
pla2-null	mean	8.160	19.268	0.539	24.263	0.111	10.870	0.00553	0.110	0.085	0.013	2.334	12.800	0.780			11.835	0.0475	0.0526	0.0460	0.0550	0.0503	6.0%	69.3%	24.8%
176 pseudopods,	95% CI	0.408	1.670	0.042	2.562	0.013	1.734	0.00017	0.053	0.018	0.004	0.070	0.384	0.016			1.059	0.0056	0.0032	0.0040	0.0309	0.0055	1.4%	8.1%	4.0%
12 cells, 3 movies	n	194	194	194	182	14	14	19	194	194	194	194	194	4707			14	14	19	194	194	19	194	194	194
gc-null	mean	4.640	10.651	0.526	11.169	0.245	4.213	0.01809	0.011	0.165	0.021	2.483	3.216	0.948			3.714	0.0987	0.0951	0.0832	0.0958	0.0932	0.6%	77.1%	22.3%
152 pseudopods,	95% CI	0.231	0.756	0.068	1.187	0.015	0.818	0.00044	0.045	0.043	0.008	0.135	0.040	0.031			0.429	0.0080	0.0046	0.0059	0.0354	0.0067	1.2%	10.3%	4.3%
4 cells, 2 movies	n	152	152	152	148	13	13	17	152	152	152	152	152	1697			13	13	17	152	152	17	152	152	152
gbpC-null	mean	5.022	9.307	0.636	9.460	0.270	3.883	0.02350	0.016	0.160	0.016	2.247	3.426	0.983			3.655	0.1201	0.1084	0.0952	0.1054	0.1073	1.1%	83.5%	15.5%
163 pseudopods,	95% CI	0.273	0.742	0.045	1.127	0.028	0.946	0.00081	0.061	0.026	0.011	0.150	0.190	0.035			0.568	0.0148	0.0074	0.0076	0.0443	0.0093	2.0%	7.6%	5.4%
4 cells, 2 movies	n	163	163	163	159	12	12	13	163	163	163	163	163	1541			12	12	13	163	163	13	163	163	163
myoII-null	mean	5.015	13.612	0.424	14.631	0.121	1.706	0.01392	0.002	0.128	0.026	1.655	1.940	0.932			1.823	0.0730	0.0834	0.0651	0.0711	0.0732	0.1%	64.5%	35.4%
170 pseudopods,	95% CI	0.215	0.750	0.032	1.562	0.012	0.367	0.00056	0.100	0.036	0.010	0.290	0.675	0.030			0.521	0.0148	0.0068	0.0036	0.0463	0.0089	2.1%	9.3%	6.6%
4 cells, 2 movies	n	170	170	170	166	14	14	13	170	170	170	170	170	2487			14	14	13	170	170	13	170	170	170
Rap1G12V	mean	3.064	10.400	0.351	9.205	0.200	1.434	0.011	0.389	0.173	0.032	3.104	1.652	1.543			1.489	0.0645	0.0747	0.0852	0.0950	0.0799	13.7%	53.3%	33.0%
150 pseudopods,	95% CI	0.159	0.780	0.029	1.112	0.014	0.389	0.002	0.170	0.025	0.008	0.085	0.030	0.099			0.244	0.0048	0.0202	0.0064	0.0621	0.0117	3.0%	4.3%	4.4%
4 cells, 2 movies	n	150	150	150	146	12	12	13	150	150	150	150	150	700			12	12	13	150	150	13	170	170	170
forAEH-null	mean	3.490	13.396	0.280	6.990	0.210	1.353	0.01196	0.270	0.164	0.024	3.614	1.431	1.928			1.392	0.0582	0.0773	0.0662	0.0671	0.0672	7.8%	59.1%	33.1%
106 pseudopods,	95% CI	0.226	0.935	0.018	1.241	0.017	0.148	0.00076	0.113	0.039	0.008	0.520	0.115	0.083			0.132	0.0096	0.0098	0.0046	0.0347	0.0073	1.6%	7.3%	5.5%
6 cells, 4 movies	n	106	106	106	100	11	11	8	106	106	106	106	106	740			11	11	8	106	106	8	106	106	106
racE-null	mean	4.199	11.193	0.433	5.030	0.231	1.226	0.01435	0.320	0.189	0.016	2.914	1.165	2.158			1.196	0.0792	0.0847	0.0792	0.1007	0.0859	12.5%	71.4%	16.1%
140 pseudopods,	95% CI	0.242	0.944	0.031	0.999	0.034	0.204	0.00043	0.128	0.050	0.005	0.475	0.106	0.115			0.155	0.0175	0.0050	0.0067	0.0609	0.0113	2.5%	9.9%	2.6%
6 cells, 4 movies	n	140	140	140	134	14	14	14	140	140	140	140	140	704			14	14	14	140	140	14	140	140	140
lrrA-null	mean	4.348	7.889	0.724	7.114	0.266	1.249	0.02927	0.140	0.157	0.027	1.280	1.120	1.108			1.185	0.2082	0.1210	0.1123	0.1387	0.1451	10.2%	68.5%	21.4%
178 pseudopods,	95% CI	0.255	0.696	0.092	0.939	0.044	0.194	0.00121	0.068	0.035	0.012	0.130	0.100	0.052			0.147	0.0404	0.0100	0.0099	0.0600	0.0150	2.6%	8.2%	5.0%
6 cells, 2 movies	n	178	178	178	172	7	7	22	178	178	178	178	178	1266			7	7	22	178	178	22	178	178	178
Neutrophils	mean	3.319	5.149	0.751	7.859	0.229	3.478	0.06726	0.078	0.250	0.023	1.287	4.930	0.661	1.772	37%	4.204	0.1784	0.1834	0.1721	0.1978	0.1829	5.8%	82.5%	11.7%
150 pseudopods,	95% CI	0.205	0.449	0.058	1.073	0.026	0.752	0.00439	0.046	0.064	0.013	0.025	0.025	0.034	0.113	3%	0.389	0.0204	0.0240	0.0150	0.0627	0.0153	1.7%	11.2%	3.4%
8 cells, 2 movies	n	150	150	150	142	14	14	9	150	150	150	150	150	1178	162	162	14	14	9	150	150	9	150	150	150
Mesenchymal cells	mean	13.797	461.214	0.0467	1289.1	0.00128	3.201	0.000018	0.020	0.070	0.00001	0.545	5.260	0.382	3.379	11%	4.231	0.00235	0.00301	0.00192	0.00292	0.00255	0.1%	99.4%	0.5%
140 pseudopods,	95% CI	0.980	70.328	0.0056	206.1	0.00013	0.511	0.000002	0.095	0.009	0.00010	0.010	0.020	0.025	0.212	1%	0.266	0.00025	0.0007	0.00029	0.00448	0.0007	0.2%	9.9%	2.4%
10 cells, 2 movies	n	140	140	140	130	7	7	6	140	140	140	140	140	1640	95	95	7	7	6	140	140	6	140	140	140
B.d. chytrid	mean	2.210	5.110	0.510	7.262	0.400	5.284	0.07860	0.080	0.319	0.060	1.829	6.243	0.750	1.702	44%	5.764	0.2187	0.1982	0.1734	0.2055	0.1990	3.9%	67.0%	29.2%
138 pseudopods,	95% CI	0.128	0.444	0.042	0.767	0.023	0.963	0.00119	0.031	0.081	0.019	0.055	0.450	0.046	0.144	5%	0.706	0.0143	0.0060	0.0151	0.0499	0.0107	0.8%	9.0%	4.8%
4 cells, 2 movies	n	138	138	138	134	14	14	15	138	138	138	138	138	427	84	84	14	14	15	138	138	15	138	138	138
source mean		measurements: mean and SD				linear regression (Fig. 2B): mean		lin regr (Fig. 4B): mean	non-linear fitting and bootstrap equation S10: mean and SDb (SD of bootstrap)			non-lin fitt and bootstrap eq S17/18: mean and SDb		measurements: mean and SD			mean	using obtained para-meters ** $\tilde{\beta} = 0.5\sqrt{\pi}$(kt+ks*v)/(1-kv*v)				mean	using obtained parameters and equation S8: (kv*v+ks*s+kt*t=1)		
source 95% CI		1.96*SD/sqrt(n)				linear regression (Fig. 2B): 95% CI		lin regr (Fig. 4B): 95% CI	1.96*SDb			1.96*SDb		1.96*SD/sqrt(n)			mean					mean/ sqrt(4)			
source n		pseudopods			pseu-cells	time points in linear regression		time points	pseudopods			pseudopods		frames									pseudopods		

Color code significance

- (orange) not significantly different from zero at P>0.1
- (green) significantly different from polarized *Dictyostelium* WT at P<0.001, and effect large (>1.5-fold, thus below 66% or above 150% of WT)
- (light blue) statistically significant different from polarized Dictyostelium WT at P<0.001 , but effect small (between 66% and 150% of WT)
