# Supplementary material for: Unified control of amoeboid pseudopod extension in multiple organisms by branched F-actin in the front and parallel F-actin/myosin in the cortex
Source: PLoS One. 2020 Dec 9;15(12):e0243442. doi: 10.1371/journal.pone.0243442 (PMC7725310; doi:10.1371/journal.pone.0243442)
Supplement: S1 File — (PDF) [file pone.0243442.s005.pdf]

Supporting information

Supporting information to: Unified control of amoeboid pseudopod extension in multiple organisms by branched F-actin in the front and parallel F-actin/myosin in the cortex, by Peter J.M. van Haastert

Supporting information contains:
Figures S1 to S7.
Text S1. Theoretical background of the start and stop of pseudopod extension.
Table S1. Source of movies.
Table S2. Overview of all pseudopod data
Movies S1. Movie 1 and 2

Supporting figures

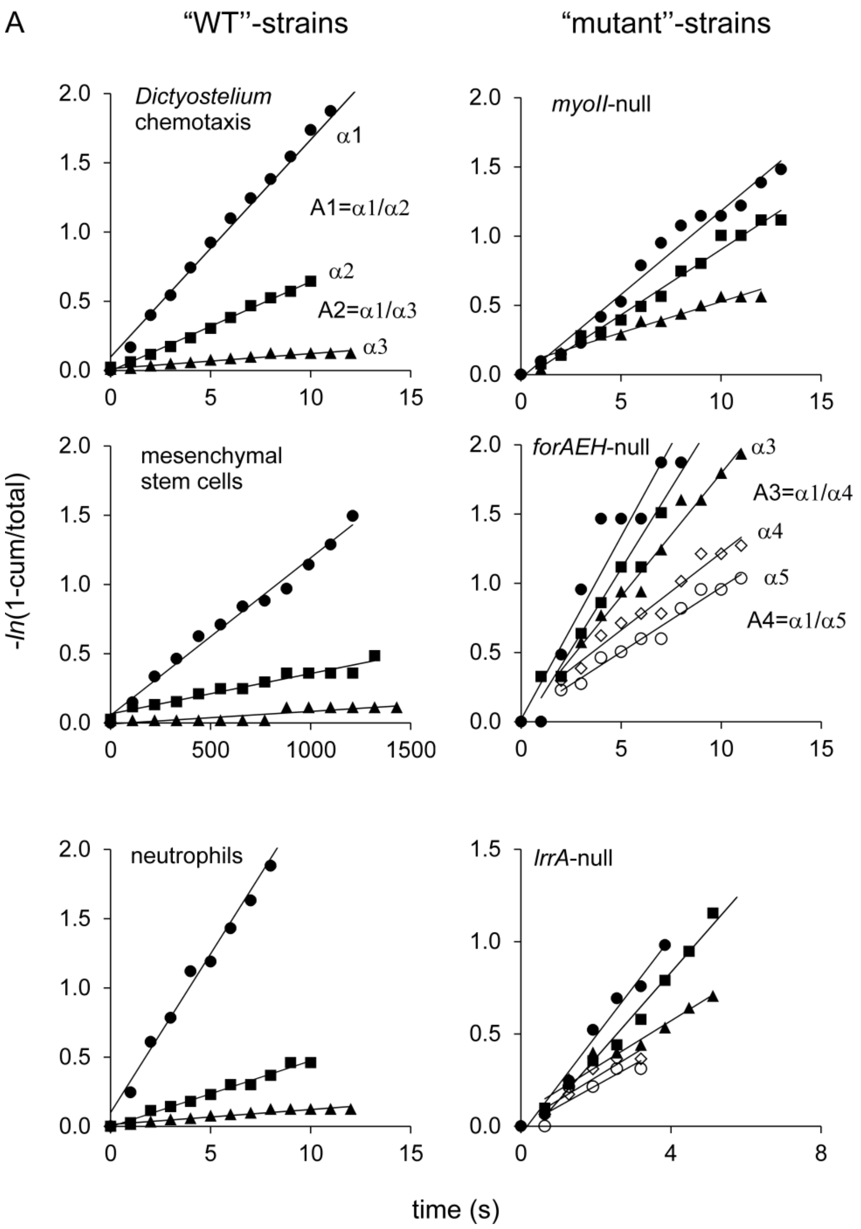

B

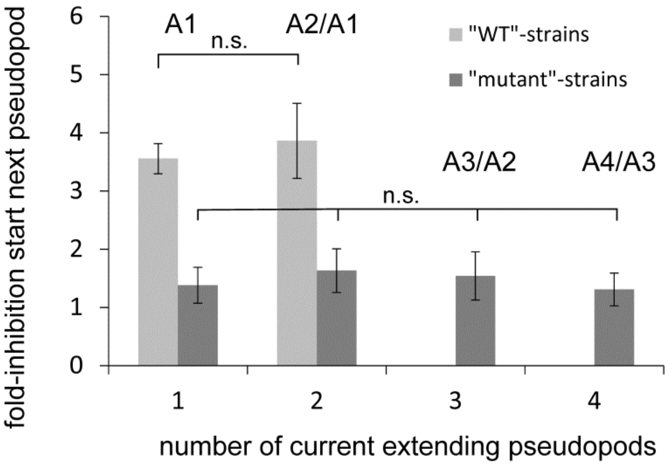

C

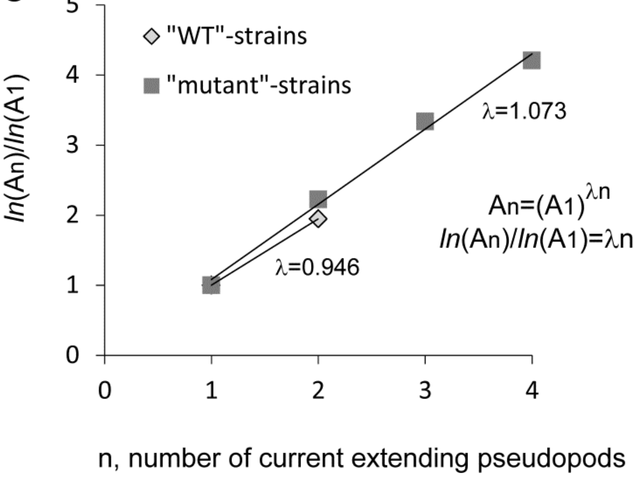

S1 Fig. Meta-analysis of the inhibition of pseudopod START by extending pseudopods.
Experiments presented in Fig 2 reveal that the START of a pseudopod in a cell that has n-extending pseudopods is inhibited A_n-fold; for polarized Dictyostelium cells A_1 = 3.5; A_2 = 13. Here the data are combined for strains with “WT” properties (7 strains: polarized AX3, unpolarized AX3, chemotaxis AX3, scar-null, neutrophils, mesenchymal cells and *B.d. chytrid*) and for strains with “mutant” properties (5 strains: *myoII*-null, *forAEH*-null, *racE*-null , *lrrA*-null, and Rap1G12V). Panel A presents the source data for three “WT” strains and three “mutant” strains. The slope αn represents the rate constant for the start of the nth pseudopod. Panel B presents the values A_n/A_{n-1}, which are not significantly different for different values of n. The data shown are the means and SEM. Panel C tests the hypothesis that $A_n = A^n$, i.e. each extending pseudopod inhibits the START of a new pseudopod by a factor A. The slope $\lambda \approx 1$ confirms the hypothesis.

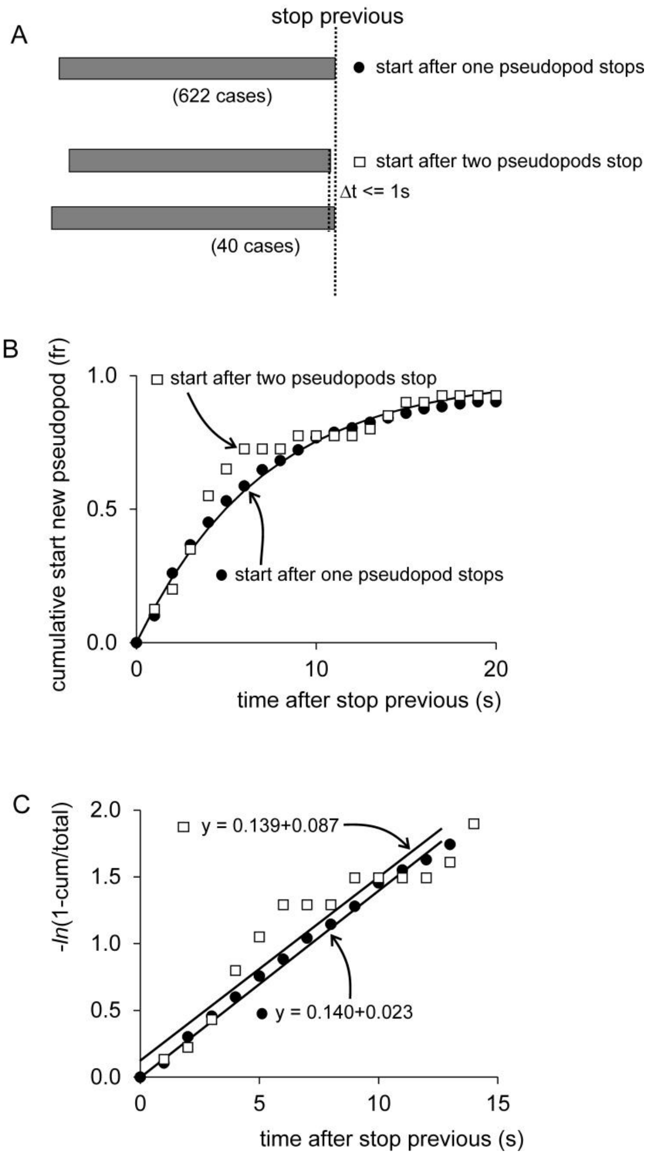

S2 Fig. The start of pseudopods after two pseudopods stop simultaneously. (A). The database of 996 pseudopods of polarized Dictyostelium cells contains 622 cases where one pseudopod stops and no other pseudopods are present. The start of new pseudopods was analyzed in Fig 2. The database of 1754 pseudopods from wild-type cells at different conditions (polarized, non-polarized and chemotaxis) contained 40 cases in which cells have two extending pseudopods that stop nearly simultaneously either in the same frame or with one frame difference. (B) Cumulative fraction of pseudopods that have started after the previous one pseudopod stopped (closed circles) or the previous two pseudopods stopped simultaneously (open boxes). (C) Kinetic analysis. Linear regression yield a slope of 0.140 ± 0.005 s^{-1} for one pseudopod stopped and a slope of 0.136 ± 0.019 s^{-1} for two pseudopod stopped (best fit and 95% CI; slopes are not significantly different; t-test). The intersection with the time axis (t_0) is -0.17 ± 0.27 s for one pseudopod stopped and -0.67 ± 1.22 s for two pseudopod stopped (these data are not significantly different from each other and also not significantly different from zero seconds, t-test). The results show that the strong 13-fold inhibition of the start of a new pseudopod by two extending pseudopods is released immediately upon stop of these two pseudopods.

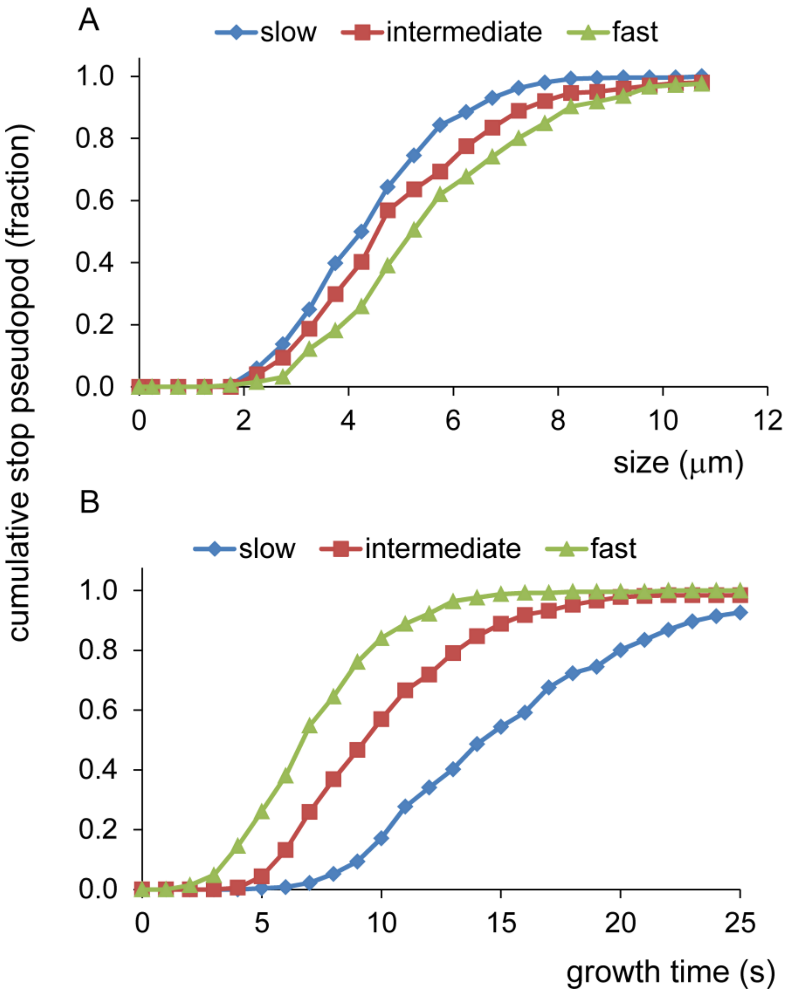

.

S3 Fig. The role of size and time in stopping of a pseudopod. The 996 pseudopods were subdivided into three groups, depending on their rate of extension: slow, intermediate and fast pseudopods. Pseudopods were binned in size intervals or growth time intervals, and the cumulative number of pseudopods at increasing size (A) or increasing time (B) was calculated. The figures show that slow, intermediate and fast pseudopod have similar size-dependent but very different time-dependent kinetics, indicating that pseudopod STOP is mainly mediated by the size of pseudopod and not by time of extension.

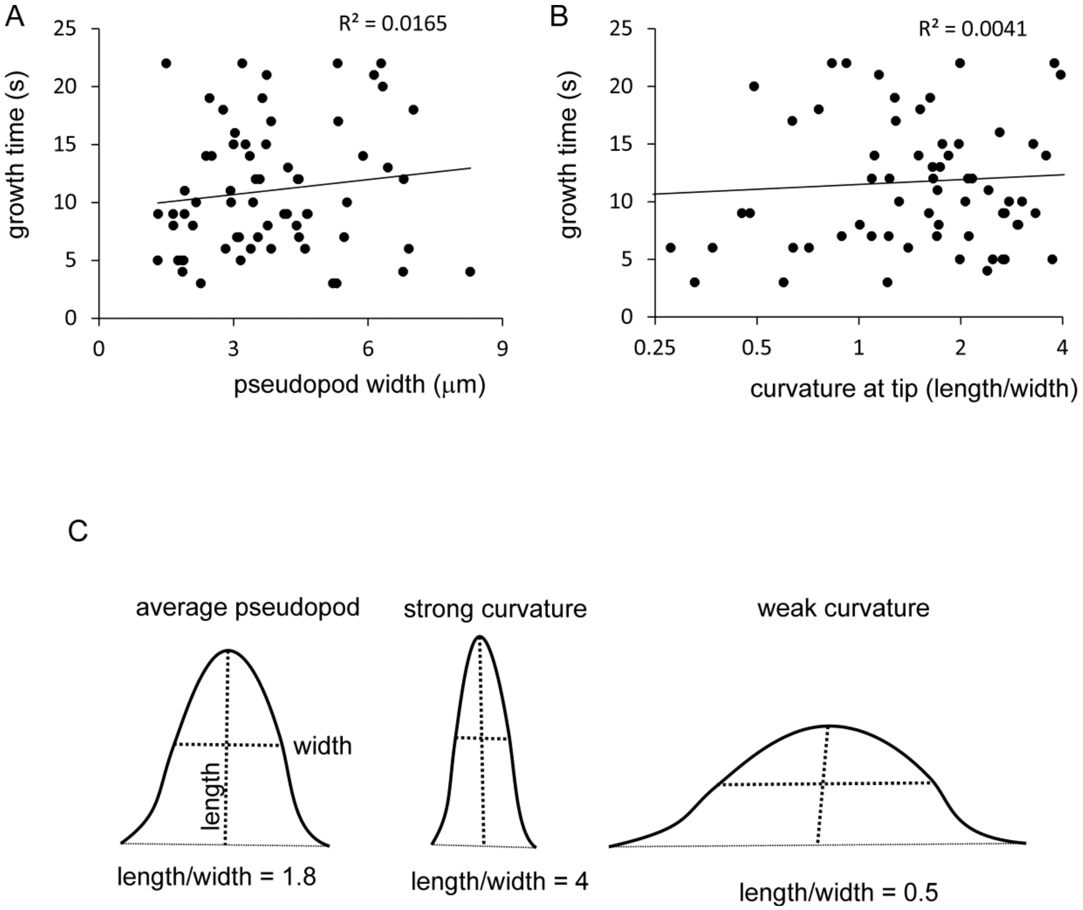

S4 Fig. The pseudopod growth time does not depend on pseudopod width or curvature. The algorithm to identify pseudopods generates information on the growth time, length and width of the extending pseudopod. The length/width ratio is an indication of the curvature of the pseudopod at the tip as shown in panel C. Pseudopod width and curvature appear to have no detectable effect on the pseudopod growth time.

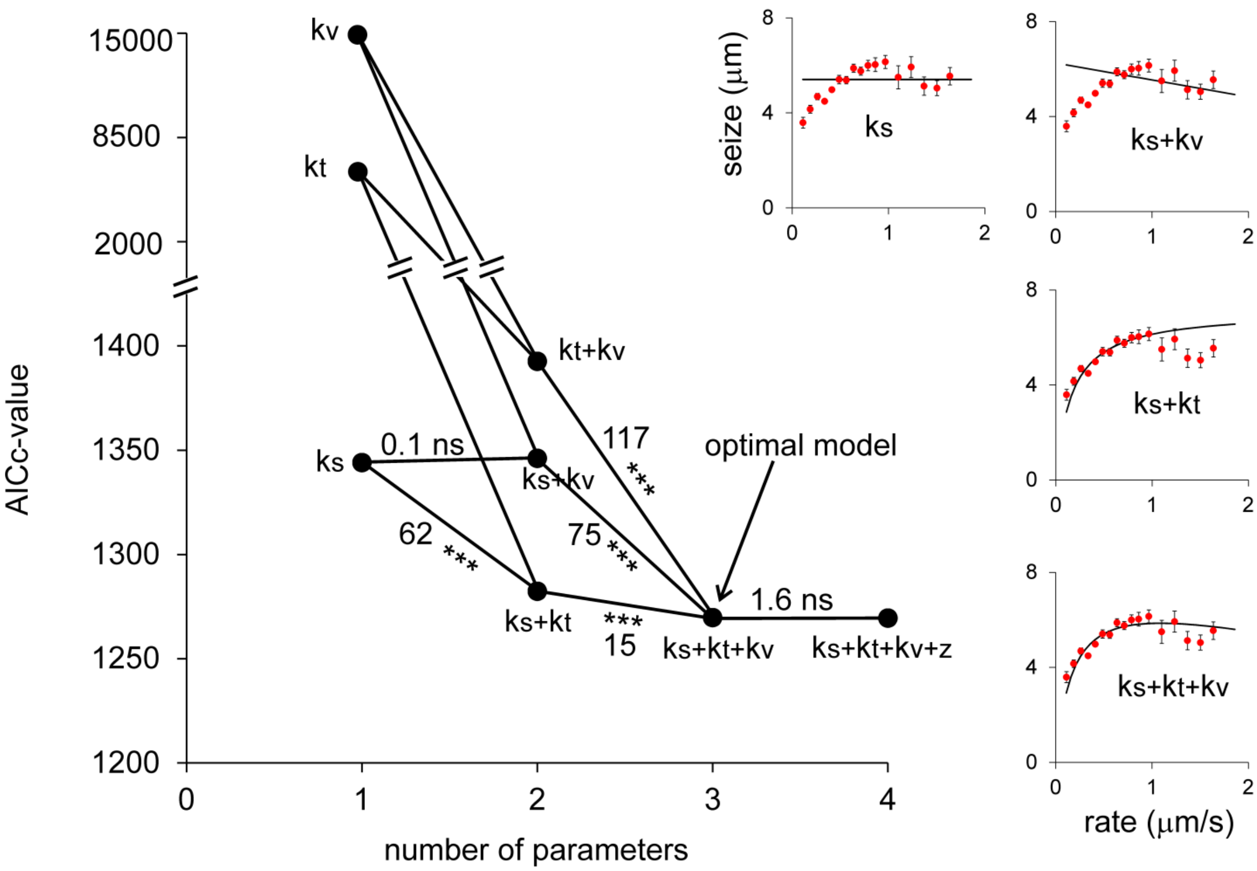

S5 Fig. Model discrimination of STOP. The size of 996 pseudopods as a function of their extension rate (Fig 4E) were fitted to equation 1 of the main text (Equation S10 in the S1 Text below) for different number of parameters, k_s, k_t and k_v. The parameter values yielding the lowest residual sum of squares (RSS) is the optimal fit. The data were analyzed with all combinations of one, two or three parameters, see the small figures for the four best fits with one, two or three parameters. Main figure: All models were evaluated with the Akaike Information Criterion (AICc) and the F-test (see S1 Text for details). The model with the lowest AICc value is the preferred model. The numbers at the line segments connecting two models is the F-value for the difference of the two model indicated; ***, the difference is significant at P<0.001; ns, not significant at P>0.2. This analysis reveals that the model with three parameters is the optimal model. Additional models with four parameters were tested, such as adding an exponent z in $k_v^z v$ or $k_v v^z$, but the slightly lower RSS was not significant by AICc and F-test to accept models with four parameters.

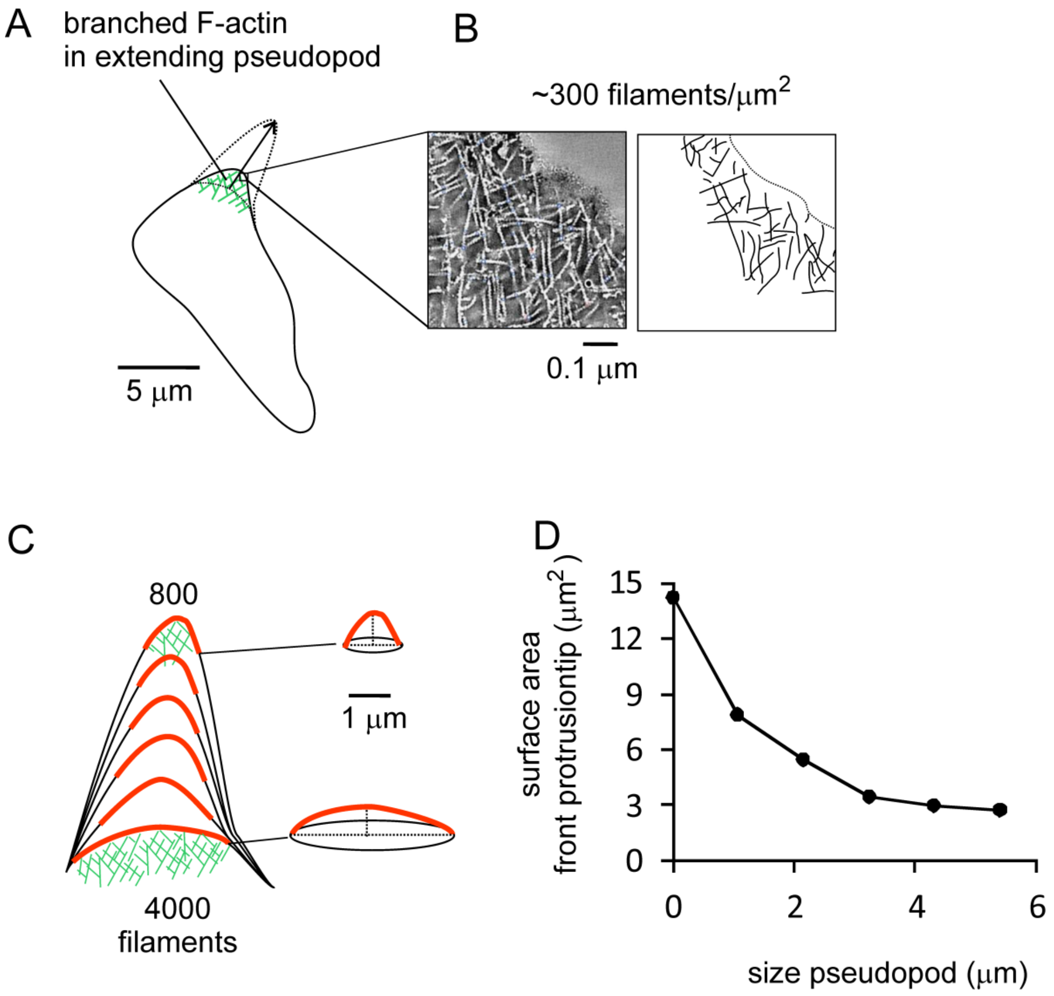

S6 Fig. Estimate of the number of branched F-actin filaments in extending pseudopods. (A) Schematic of a cell with an extending pseudopod; the dashed lines are the boundary of the cell at the start and stop of pseudopod extension, respectively. (B) Left; tomography image of a protrusion (Figure 3a from Urban et al. 2010 Nat Cell Biol.12:429–35); Right; drawing of filaments near the boundary. The density of actin filaments at the boundary of the protrusion is estimated to be about 300 filaments/μm^2 and the layer of branched F-actin in the protrusion is about 1 µm thick. (C) Geometry of a representative extending pseudopod of *Dictyostelium*. Black lines are the boundary of the extending pseudopod at different sizes, while red lines indicate the front of the protrusion up to 1 µm from the top, representing the region of branched F-actin. The two images at the right represent the three-dimensional shape of the front of the protrusion. (D) The outer surface area of the front of the protrusion up to 1 µm from the tip (the red dome in panel C) was estimated for 5 pseudopod of polarized *Dictyostelium* cells; the surface area declines from 14 μm^2 at the start of pseudopod extension to about 3 μm^2 at the end of pseudopod extension. This decline of surface area at the tip is expected to be associated with a reduction of branched F-actin in the leading pseudopod from about 4000 actin filaments at the start of extension to about 800 actin filaments at the end of extension.

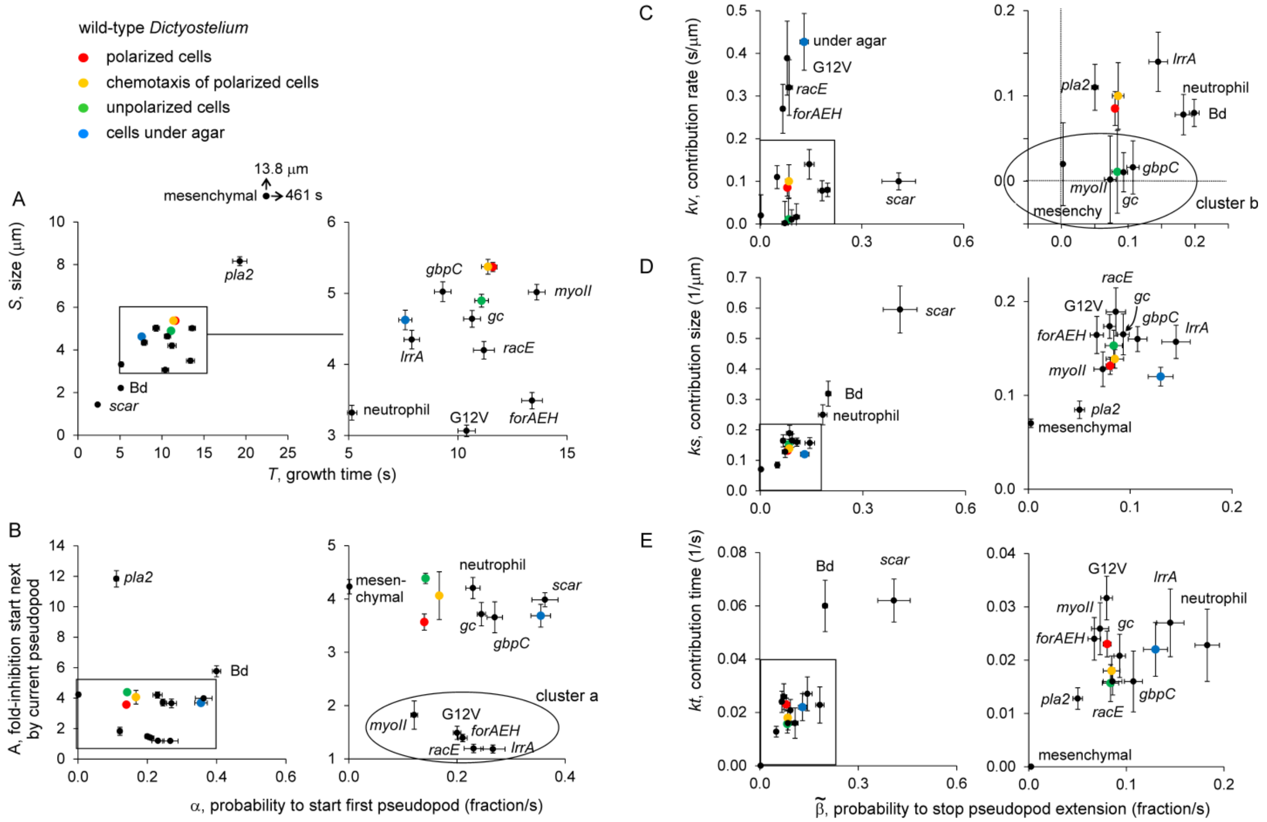

S7 Fig. Pseudopod extension at different conditions, mutants and organisms. Pseudopod extension was measured in wild-type *Dictyostelium* cells at four different conditions (colored symbols), in nine *Dictyostelium* mutants, and in three other organisms, human neutrophils, human mesenchymal stem cells and fungal *B.d. chytrid* (B.d.). The data show the means and SEM. The boxed area in the left panels is enlarged in the right panels. In panel A, mesenchymal cells are far out of scale as indicated. For all panels, polarized cells (red symbol) and chemotaxis (yellow symbol) are statistically not different in any respect. Panel C reveals that unpolarized cells (green symbol) specifically lack the influence of the rate on pseudopod STOP (k_v), while cells under agars exhibit a strongly increased effect of the rate on STOP (k_v). Mutant cells are described individually in the results. Cluster **a** in panel B are mutants in which an extending pseudopod hardly inhibits the extension of a new pseudopod; these mutants are all defective in the regulation of a strong parallel pF-actin cell cortex (see Fig 6F). Cluster **b** in panel C are mutants in which STOP is not affected by the rate of pseudopod extension; these mutants all lack a strong uropod (see Fig 6G). Neutrophils are similar in many respects to *Dictyostelium* cells. *B.d. chytrid* extend pseudopods at a relatively high rate that stop at a small size, but otherwise many properties are similar to *Dictyostelium* and neutrophils. Although on first sight mesenchymal cells are very different with a very low extension rate and very long growth times, many other aspects are very similar, including inhibition of new pseudopods by current pseudopods and stopping by mainly size.

S1 Text

Theoretical background of the kinetics of pseudopod extension.

Supporting material to *Unified control of amoeboid pseudopod extension in multiple organisms by branched F-actin in the front and parallel F-actin/myosin in the cortex* by Peter J.M. van Haastert

A. Introduction

The pseudopod starts or stops abruptly; in between start and stop, the pseudopod extends at a constant rate (Fig. 1B). Therefore, pseudopod extension is regulated by a binary switch between 0 (no extension) and 1 (extending). A pseudopod starts when somewhere in the cell a pseudopod-inducing activity surpasses a threshold and pseudopod activity switches from 0 to 1. As long as the pseudopod is extending, the pseudopod activity is 1. The pseudopod stops because pseudopod activity switches from 1 to 0.

We have investigated the probability that a cell starts or stops pseudopod extension as a function of i) the presence of other pseudopods, ii) random versus regulated, and iii) time, size and rate of pseudopod extension. The current analysis reveals that the kinetics of start and stop events are not random, but regulated in a complex way depending on number and size of pseudopods. Since start and stop are not random events, also the distribution of cells with different number of pseudopods is not based on random events.

Here the theoretical background is provided for the analysis of start and stop of pseudopod extension, and of the distribution of cells with different numbers of pseudopods.

A cell may extend pseudopods according to the general scheme below:

$$C_0 \underset{\beta_1}{\overset{\alpha_1}{\rightleftarrows}} C_1 \underset{2\beta_2}{\overset{\alpha_2}{\rightleftarrows}} C_2 \underset{3\beta_3}{\overset{\alpha_3}{\rightleftarrows}} C_3 \rightleftarrows \quad \underset{n\beta_n}{\overset{\alpha_n}{\rightleftarrows}} C_n \rightleftarrows \qquad \text{Scheme 1}$$

Here C_n denotes a cell with n pseudopods, α_n is the probability to start the nth pseudopod and β_n is the probability of stopping of one of its n pseudopod.

B. The START of a pseudopod

Experiments reveal that in cells without pseudopods the start of the first pseudopod is stochastic. In addition, the start of a second pseudopod is also stochastic, but is inhibited by the first pseudopod, while the start of a third pseudopod is inhibited even further (Fig. 2). This inhibition appears not to depend on the distance from the extending pseudopod (1). Furthermore, at the time-resolution of the experiments (1s) the inhibition appears and disappears virtually immediate after a pseudopod starts and stops, respectively (Fig. 1C). This suggests that an extending pseudopod produces and inhibitor that -without a delay and in the entire cell- reduces the probability to start a new pseudopod. Therefore, the rate constant for start of a new pseudopod α_n depends on the number of pseudopods already present.

B.1. Equations for the START of a pseudopod
In a cell that has *n-1* extending pseudopods (E_{n-1}), the start of the extension of the nth pseudopod (E_n^+) is given by

$$\frac{dE_n^+(t)}{dt} = \alpha_n E_{n-1}(t) \qquad \text{(S1)}$$

For the start of the first pseudopod (*n*=1), $E_{n-1}(t)$ in the right part of equation (S1) is given by $E_0(t) = 1 - E_1(t)$ and the equation is solved yielding

$$-\ln[1 - E_1^+(t)] = \alpha_1 t \qquad \text{(S2)}$$

For the start of a second pseudopod, initially all cells with one pseudopod are available, but during time an increasing fraction of those cells have stopped this first pseudopod and are not available anymore to extend a second pseudopod. Therefore, the fraction of cells extending a second pseudopod at time *t* has to be corrected for the reduced number of cells with one pseudopod at that time. With this correction the differential equation is solved yielding

$$-\ln[1 - \frac{E_2^+(t)}{E_1(t)}] = \alpha_2 t \qquad \text{(S3)}$$

where $E_2^+(t)$ is the fraction of cells that have started a second pseudopod at time *t*, and $E_1(t)$ is the fraction of cells in which the first pseudopod is still extending at time *t*. Care was taken that the analysis was not strongly influenced by the reduced fraction of cells with one pseudopod $E_1(t)$; therefore the start of the second pseudopod was measured up to 13 seconds at which at least 30% of the first pseudopods are still extending.

In a similar way the start of a third pseudopod was analyzed with correction for the reduced number of cells that have still both pseudopods extending, yielding

$$-\ln[1 - \frac{E_3^+(t)}{E_2(t)}] = \alpha_3 t \qquad \text{(S4)}$$

B.2. Data analysis of the START of a pseudopod
In a large database of 622 cells that have no pseudopods the start of the first pseudopod was analyzed yielding $\alpha_1 = 0.14$ s^{-1}. Then, in this set of 622 cells with one pseudopods the start of second pseudopod was analyzed yielding $\alpha_2 = 0.04$ s^{-1}. And in the set of 277 cells with two pseudopods the start of third pseudopod was analyzed yielding $\alpha_3 = 0.01$s^{-1}. From this analysis it appears that each extending pseudopod reduced the rate constant A-fold (S1 Fig):

$$\alpha_n = \frac{\alpha_1}{A^{n-1}} \qquad \text{(S5)}$$

In summary, the start of a pseudopod is described by two parameters, the stochastic rate constant α_1 of formation of the first pseudopod, and the *A*-fold inhibition of this rate constant by each extending pseudopod present.

C. The STOP of pseudopod extension

Experiments reveal that the stop of pseudopods is not influenced by the presence of other pseudopods (Fig. 3). Thus STOP is a pseudopod-self event, which can be analyzed without taking other pseudopods into account (as was critical for pseudopod START above).
Experiments also reveal that, depending on the cell lines and conditions used, the growth time, size and rate of pseudopod extension determine the stopping of the pseudopod. Here equations are derived explaining these observations. Two methods are given that were used to derive kinetic constants that describe the STOP of individual pseudopods and of the population of pseudopods, respectively; both methods are based on a general physical model of pseudopod extension.

C.1. Physical model

Extending pseudopods contain polymerizing dendritic filamentous actin, which generate a forward force/activity that is proportional to the number of polymerizing actin filaments in the tip of the pseudopod. The forward activity at the start of pseudopod extension is defined as F_0. This forward force/activity may experience several counter forces/activities (*C*). Experiments support a model in which these counteractivities are proportional to the time, size and rate of pseudopod extension (Fig. 4E). Multiple inhibitory processes may contribute to these counteractivities. Example for <u>time:</u> The extending pseudopod may produce an inhibitor that reduces the forward activity. It is assumed that the inhibitor is produced by the extending pseudopod at a constant rate, and therefore the time-dependent counterforce is given by $C_t(t) = -a_t t$. Example for <u>size:</u> The extending pseudopod may experience a counterforce due to tension, which is proportional to the size of the extending pseudopod as in a spring, i.e. $C_s(t) = -a_s s(t)$. Example for <u>rate:</u> The extending pseudopod may experience a counterforce due to drag. The drag of a small object (extending pseudopod) in a liquid at low Reynolds number is proportional to dynamic viscosity η of the liquid, the radius *R* of the object and the speed *v* of the object (2,3); therefore $C_v(t) = a_v v(t)$, where $a_v = 6\pi R\eta$. Under agar or in the extracellular matrix extending pseudopods experience a counterforce to overcome deformation of the poro- and visco-elastic agarose or extracellular matrix; this counterforce is proportional to the rate of deformation, i.e. the rate of pseudopod extension.

During pseudopod extension the rate of extension is constant (Fig 1B), i.e. $v(t) = v$. The net forward activity *F(t)* during pseudopod extension is therefore given by

$$F(t) = F_0 - C_t(t) - C_s(t) - C_v(t) = F_0 - a_t t - a_s s(t) - a_v v(t) \qquad \text{(S6)}$$

C.2.1. Equations for the STOP of individual pseudopods

At the moment a pseudopod stops it has growth time $t = T$ and size $s(t) = S$. It is observed that pseudopods extend at a constant rate and then stops. Therefore, it is assumed that a pseudopod switches between full extension and stopping when the net forward activity *F(t)* declines below a threshold value (F_r)

At the moment of pseudopod stopping the actual forward activity *F(t)* equals the threshold F_r:

$$F_t = F_0 - a_t T - a_s S - a_v v \qquad \text{(S7),}$$

which is identical to $\frac{a_t}{F_0 - F_t} T + \frac{a_s}{F_0 - F_t} S + \frac{a_v}{F_0 - F_t} v = 1$ (S7b)

The time-, size and rate-dependent parameters a_t, a_s and a_v, respectively, are normalized according $k_i = a_i/(F_0 - F_t)$, yielding

$$k_t T + k_s S + k_v v = 1 \qquad \text{(S8)}$$

Equation S8 describes the relative contribution of time, size and rate to STOP of pseudopod extension. Furthermore, the relation between pseudopod growth time and size is given by $S = v*T$. With this, equation S8 is solved for the growth time T and size S at pseudopod STOP, respectively.

$$T = \frac{1-k_v v}{k_t + k_s v} \qquad \text{(S9)}$$

$$S = \frac{1-k_v v}{k_t/v + k_s} \qquad \text{(S10)}$$

Thus, the size and growth time of a pseudopod at STOP depends on four factors: the speed v, and the time-, size- and rate-dependent parameters k_t, k_s and k_v, respectively.

C.2.2. Data analysis of the STOP of individual pseudopods

The rate-dependence of the size at STOP of 966 pseudopods was analyzed with equation S10 using the least square method, yielding the minimal RSS and the optimal parameter values for k_t, k_s and k_v. The Bootstrap method (4) was used to provide an estimate of the goodness of the fit (95% confidence interval of the parameter).

The model has three parameters. To investigate the relevance of these three, or possibly additional parameters, different models were analyzed: three models with one parameter (either k_t, k_s or k_v), three models with two parameters (either k_t and k_s, k_t and k_v or k_s and k_v), one model with the three parameter (k_t, k_s and k_v), and one model with four parameters (the three parameters k_t, k_s, k_v and an exponent z in the rate, v^z). The parameters in the equation were fitted to the 966 experimental observations using the least square method, yielding the optimal parameter value and the RSS for that model. The Akaike Information Criterion (AIC) and the F-test (5–8) were used to select the optimal model, i.e. the model with the lowest number of parameters that fit the data significantly better than models with the same or less parameters:

$AIC_c = 2p + N\,ln(RSS/N) + 2p(p+1)/(n-p-1)$

$F = [(N-p_2)/(p_2-p_1)][(RSS_1-RSS_2)/RSS_2]$

Where p is the number of parameters, N is the number of observations (966) and the subscript 1 and 2 indicate the model with less and more parameters, respectively.

For models with equal number of parameters, AIC was used (the preferred model has the lowest AIC value). The F-test was used for all other model discriminations to select the best model.

For the large experimental data set of polarized *Dictyostelium* cells (S5 Fig) the AIC and F-test clearly reveal that pseudopod STOP is explained significantly better by the model with three parameters (k_t, k_s and k_v) than by the various models one or two parameters. Although the additional fourth parameter (the power of the rate) reduces the RSS, this reduction is too small by the F-test to allow acceptance of a model with four parameters. Model discrimination therefore reveal that pseudopods stop by a combination of time, size and rate. The model with three parameters was then used for all other experimental data sets of wild-type cells at different conditions, mutant cells or other species; estimates for the parameter values and 95% confidence level were performed by least RSS and bootstrap method, respectively, as described above. All obtained parameter estimates are presented in S2 Table, and summarized in Table 1 and S7 Fig.

C.3.1. Equations for the STOP of a population of pseudopods

STOP of a population of pseudopods is described by the rate constant γ (unit fraction/s^2). As shown above, on a short time scale of seconds individual pseudopods STOP by a combination of time, size and rate of the extending pseudopod. Based on the observation that the fraction of stopped pseudopods increases with t^2 (Fig. 4C), the following simplification is used to obtain a fast and accurate estimate for γ. Time and size have by far the largest contribution to pseudopod STOP (94% for polarized wildtype cells); furthermore, size of a pseudopod increases linear with time. Therefore, as shown in Fig. 4B, the probability to STOP increases with time according

$$\frac{dE(t)}{dt} = -\gamma t E(t) \qquad \text{(S11)}$$

Integration yields

$$-\ln[E(t)] = 0.5\gamma(t^2 - t_0{}^2) \qquad \text{(S12)}$$

where t_0 is the time at which the processes leading to stopping begin (i.e. onset of the counterforces). The slope is 0.5γ, which provides the macroscopic rate constant $\tilde{\beta} = \sqrt{0.5\gamma}$ (see below in section E).

Combining S11 and S12 yields the probability to stop

$$\frac{dE(t)}{dt} = -\gamma t e^{-0.5\gamma(t^2 - t_0{}^2)} \qquad \text{(S13)}$$

C.3.2. Data analysis of the STOP of the population of pseudopods

The growth time of the large dataset of 996 pseudopods of polarized wild-type *Dictyostelium* cells was used to estimate the apparent rate constant γ of pseudopod stopping. As shown in Figure 4C, the expression of the fraction of extending pseudopods as $-\ln[E(t)]$ versus t^2 yields the expected straight line with slope 0.5γ and intercept $t_0{}^2$. The estimates for γ and the 95% confidence levels are summarized in Table 1 and S2 Table.

The physical model for pseudopod extension assumes a forward activity at the start of pseudopod extension and several counter activities that increase during pseudopod extension. In this model t_0 is the time when these counterforces begin to inhibit pseudopod extension. For the large dataset of polarized wild-type cells the obtained value for t_0^2 = - 3.33 ± 3.70 (mean and 95% confidence interval, n=21 time points), which is not significantly different from zero. For all 15 strains and conditions t_0^2 = 3.2 ± 10.6 (mean and SD), which is also not significantly different from zero. This suggests that the counterforces begin to increase immediately after the onset of pseudopod extension.

D. The steady state number of pseudopods per cell

For long time-series of moving cells the number of pseudopods/cell was determined. Experiments reveal that the start of a new pseudopod is stochastic with rate constant α, but is inhibited *A*-fold by each pseudopod present. The stop of a pseudopod does not does not depend on the presence of other pseudopods. As shown above, at a microscopic short time-scale of seconds, the stop of an extending pseudopod is a complex function of rate, size and time. However, at a macroscopic large time-scale of minutes or hours, the rate of stop is given by the macroscopic population rate constant ***steady state*** $\boldsymbol{\tilde{\beta}}$**.**

D.1. Equations describing the steady state number of extending pseudopods per cell

The fraction of cells with *n* extending pseudopods is defined as C_n. The fraction C_n will increase when a cell with *n-1* pseudopods starts a new pseudopod, or when a cell with *n+1* pseudopods stops one of its pseudopods. The probability to start a new pseudopod is stochastic with rate constant α, but is inhibited *A*-fold by each pseudopod present; thus the rate constant for the formation of C_n from C_{n-1} is given by α/A^{n-1}. A cell with *n* pseudopod has *n* possibilities to stop one of its pseudopods, thus the rate constant for the formation of C_n from C_{n+1} is given by rate constant $(n+1)\tilde{\beta}$. Therefore, the distribution of cells with different number of pseudopods is described by:

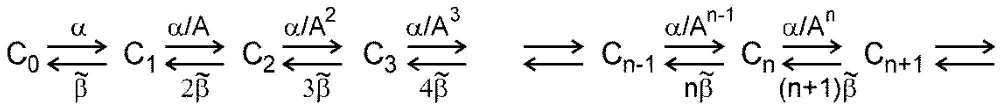

Scheme 2

The change of C_n is given by

$$\frac{dC_n}{dt} = \frac{\alpha}{A^{n-1}}C_{n-1} + (n+1)\tilde{\beta}C_{n+1} - \frac{\alpha}{A^n}C_n - n\tilde{\beta}C_n \qquad \text{(S14)}$$

Assuming a steady state for equation S16, it follows that

$$\frac{C_n}{C_{n-1}} = \frac{\alpha}{n\tilde{\beta}A^{n-1}} \qquad \text{(S15)}$$

The distribution of cells with *n* extending pseudopods is obtained using equation S15 by expressing C_1 to C_n as a function of C_0:

$$C_n = f(n).C_o, \text{ where } f(n) = (\alpha/\tilde{\beta})^n \frac{1}{\prod_1^n(n)\,.\,A^{\sum_1^{n-1}(n)}} \text{ for } n \geq 1 \qquad \text{(S16)}$$

Since the sum of all probabilities is 1, $C_0 + \sum_{n=1}^{\infty}(C_n) = 1$, providing the solution of C_0

$$C_0 = \frac{1}{1+\sum_{n=1}^{\infty}(f(n))} \qquad \text{(S17)}$$

Finally, combining (S16) with (S17) gives the set of equations for the distribution of the number of pseudopods per cell (see table below). Equation S16 reveals that the pseudopod distribution depends on two parameters, $\alpha/\tilde{\beta}$ and *A*.

D.2. Data analysis of the steady state number of extending pseudopods per cell

To determine the distribution of cells with different number of extending pseudopods, one continuous series of 996 extending pseudopods was made as follows: In pseudopod analysis, a cell was followed for about 15 minutes providing information on about 30 concerted pseudopods; the average pseudopod interval is 15.4 seconds. The 996 pseudopods of polarized wild type cells were obtained from 39 different cells. The pseudopods of cells 1 and 2 were concatenated by starting the first pseudopod of cell

2 at 15 seconds after the start of the last pseudopod of cell 1. After concatenating the pseudopods of all 39 cells, the total time is 15,356 seconds with information on the number of extending pseudopods at a resolution of 1 second.

The observed distribution of the number of extending pseudopods was analyzed with equations S16 and S17 using the least square method, yielding the minimal RSS and the optimal parameter values for $\alpha/\widetilde{\beta}$ and *A*. The confidence of these optimal parameter values was estimated by bootstrap analysis with random selection of 100 time segments of 153 seconds. Using the obtained values for $\alpha/\widetilde{\beta}$ and α, the value for the macroscopic rate constant ***steady state*** $\boldsymbol{\widetilde{\beta}}$ was calculated (units fraction/s).

The table below shows the equations for *f(n)*, as well as the calculated and observed fraction *C(n)* of cells with *n* extending pseudopods for polarized *Dictyostelium* cells.

n (extending pseudopods)	Equation for *f(n)*	calculated for $\alpha/\widetilde{\beta}$=1.79; A=3.55		observed	
		f(n)	*fraction C(n)*	*time (s)*	*fraction C(n)*
0			0.3065	4702	0.3062
1	$\alpha/\widetilde{\beta}$	1.79	0.5486	8705	0.5669
2	$(\alpha/\widetilde{\beta})^2 \frac{1}{2A}$	0.4513	0.1383	1843	0.1200
3	$(\alpha/\widetilde{\beta})^3 \frac{1}{6A^3}$	0.0213	0.0065	100	0.0065
4	$(\alpha/\widetilde{\beta})^4 \frac{1}{24A^6}$	2.1E-4	6.55E-5	6	3.9E-4
5	$(\alpha/\widetilde{\beta})^5 \frac{1}{120A^{10}}$	4.8E-7	1.48E-7	0*	0*
sum *f(n)*		2.2628	1	15,356	1

*Cells with 5 or more pseudopods were not observed

E. Connection between STOP kinetics and steady state number of extending pseudopods

The values for the macroscopic rate constant ***steady state*** $\tilde{\boldsymbol{\beta}}$ were obtained from the number of extending pseudopods in section D. The macroscopic rate constant $\tilde{\beta}$ can also be obtained from the kinetics of pseudopod STOP by three methods. First, from Equation S12 the macroscopic rate constant $\tilde{\beta}$ was derived:

$$\tilde{\beta} = \sqrt{0.5\gamma} \qquad \text{(S18)}$$

Second, the mean growth time T is given by the probability to stop at time t (S13), multiplied by that time t, and then summated over all times: $T = \int \gamma t^2 e^{-0.5\gamma t^2}$ which is solved to yield

$$T = 0.5\sqrt{\pi/\gamma} \qquad \text{(S19)}$$

Combining equations S18 and S19 yields

$$\tilde{\beta} = 0.5\sqrt{\pi}/T \qquad \text{(S20)}$$

Third, combining equation S20 and S9 yields

$$\tilde{\beta} = 0.5\sqrt{\pi}\frac{k_t + k_s v}{1 - k_v v} \qquad \text{(S21)}$$

This equation describes the macroscopic rate constant of stopping as a function of stopping forces that depend on the time, size and rate of pseudopod extension.

Equations S18, S20 and S21 were inspected. The experimental data on the distribution of pseudopods yield the values for the ***steady state*** $\tilde{\boldsymbol{\beta}}$, which is presented relative to the right parts of equations S18, S20 or S21 in the Figure S8 below. The slopes are close to 1 and the intercepts are close to 0, confirming equations S18, S20 and S21. This implies that the macroscopic rate constant $\tilde{\beta}$ can be obtained by four independent methods yielding similar values; in main table 1, the average of these four values is presented.

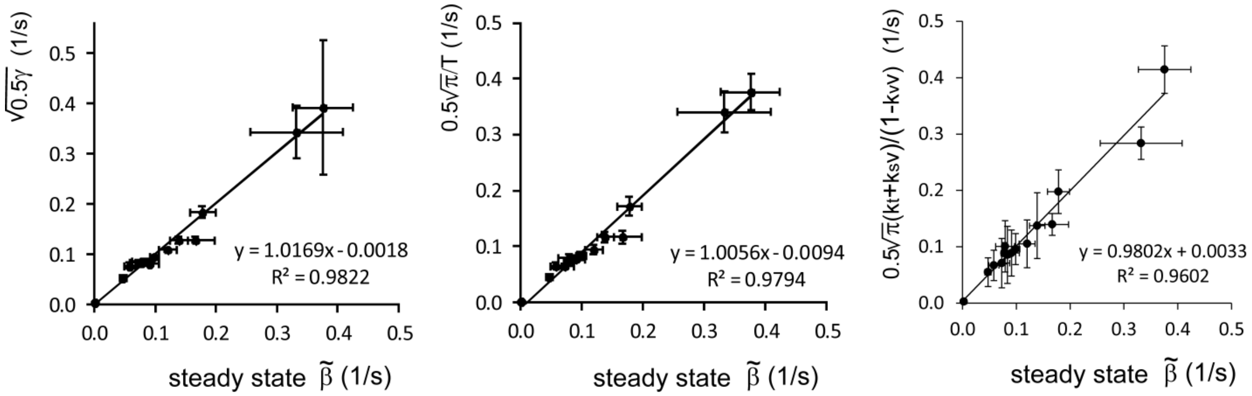

Figure S8. Experimental data from 15 cell lines and mutants of ***steady state*** $\tilde{\boldsymbol{\beta}}$ and for $\sqrt{0.5\gamma}$ (equation S18, left panel), for $0.5\sqrt{\pi}/T$ (equation S20, middle panel) and for $0.5\sqrt{\pi}\frac{k_t + k_s v}{1 - k_v v}$ (equation S21, right panel). The error bars are 95% confidence intervals.

F. References

1. Bosgraaf L, Van Haastert PJM. The ordered extension of pseudopodia by amoeboid cells in the absence of external cues. PLoS One. 2009 Jan;4(4):e5253.
2. Van Haastert PJM. Amoeboid cells use protrusions for walking, gliding and swimming. PLoS One. 2011;6(11):e27532.
3. Dusenbery D. Living at Micro Scale: The Unexpected Physics of Being Small. Cambridge, Mass: Harvard University Press; 2009.
4. Hu W, Xie J, Chau HW, Si BC. Evaluation of parameter uncertainties in nonlinear regression using Microsoft Excel Spreadsheet. Environ Syst Res. 2015;4(1):4.
5. Golla SS V, Adriaanse SM, Yaqub M, Windhorst AD, Lammertsma AA, van Berckel BNM, et al. Model selection criteria for dynamic brain PET studies. EJNMMI Phys. 2017 Dec;4(1):30.
6. Hurvich CM, Tsai CL. Model selection for extended quasi-likelihood models in small samples. Biometrics. 1995 Sep;51(3):1077–84.
7. Ludden TM, Beal SL, Sheiner LB. Comparison of the Akaike Information Criterion, the Schwarz criterion and the F test as guides to model selection. J Pharmacokinet Biopharm. 1994 Oct;22(5):431–45.
8. van Haastert PJ, de Wit RJ, Janssens PM, Kesbeke F, DeGoede J. G-protein-mediated interconversions of cell-surface cAMP receptors and their involvement in excitation and desensitization of guanylate cyclase in Dictyostelium discoideum. J Biol Chem. 1986 May;261(15):6904–11.

Supporting movies

S1 movies. *Dictyostelium* cells express the dominant active RapAG12V from an inducible promotor, and express the sensors Ral-GDS-GFP and cytosolic-RFP. The movie is recorded at 4 seconds per frame.
Movie1 <RapAG12V_phase contrast> shows the phase contrast of three cells moving in buffer.
Movie 2 < RapAG12V_Ral-GDS> shows the calculated Ψ for the same three cells. Ψ is a sensitive assay for Rap1-GTP levels and was calculated for each pixel *i* as described in (28): $\Psi_i=(GR_i-cRD_i)/<GR_{cyt}>$, where GR is the intensity of the GFP channel, cRD is the intensity of the RFP channel, and $<GR_{cyt}>$ is the mean intensity of the GFP channel in large areas of the cytosol.
