# Supplementary material for: Unified control of amoeboid pseudopod extension in multiple organisms by branched F-actin in the front and parallel F-actin/myosin in the cortex
Source: PLoS One. 2020 Dec 9;15(12):e0243442. doi: 10.1371/journal.pone.0243442 (PMC7725310; doi:10.1371/journal.pone.0243442)
Supplement: Attachment — Submitted filename: Response to Reviewers.docx [file pone.0243442.s006.docx]

Reviewer #1: I'm happy with the changes made to this paper. I'm also happy the formin inhibitor was not used, given the doubts about its specificity. Delighted to see this paper published.

Thank you

Reviewer #2: For the most part, I am satisfied with the response to my comments. However, I would still like to see some individual traces of vectorial rate vs. time in addition to the average trace in Figure 1B. This would help readers assess whether individual pseudopods behave as described by the bulk data. Unless I am mistaken, the author's previous papers do not contain such a plot. I feel this request is reasonable because it would not require any additional experiments and should be simple from an analysis standpoint.

I have included the data for the vectorial rate of five individual pseudopods in Figure 1B. The legend to the figure has been changes accordingly.
